# Third molars and dental crowding: different opinions of orthodontists and oral surgeons among Italian practitioners

**DOI:** 10.1186/s40510-014-0060-y

**Published:** 2014-11-22

**Authors:** Michela Gavazzi, Donato De Angelis, Sergio Blasi, Paolo Pesce, Valentina Lanteri

**Affiliations:** Department of Biomedic, Surgery and Dentistry, School of Orthodontics, Via della Commenda 10, 20122, University of Milan, Milan, Italy; Department of Surgical and Diagnostic Sciences (DISC), School of Dentistry, University of Genoa, Largo R. Benzi 10, Ospedale San Martino, Genoa, 16132 Italy

**Keywords:** Third molar, Crowding, Extraction

## Abstract

**Background:**

The role of third molars as a cause of incisor crowding, especially in the lower arch, continues to be controversial. The aim of this work is to compare opinions of Italian oral surgeons and orthodontists on this topic.

**Methods:**

One hundred ninety-three Italian practitioners of the Society of Orthodontics (SIDO) and the Italian Society of Oral Surgery (SICOI) were asked to fill out an online questionnaire made up of six questions. Practitioners were asked to express their opinion on the relation between upper and lower third molar eruption and anterior crowding.

**Results:**

One hundred sixty-six members of both societies completed the online research survey; response rate (RR) was 86%. There were no statistically significant differences between the two groups (*P* > 0.005). Both agree not to believe that third molars create a force responsible for anterior crowding in the upper (82.5% orthodontists, 83.8% surgeons) and in the lower arch (52.6% orthodontists, 63.8% surgeons). Both agree also not to consider the upper (89.7% orthodontists, 82.1% surgeons) and lower (58.8% orthodontists, 63.2% surgeons) third molar extraction useful to prevent crowding.

**Conclusions:**

Italian orthodontists and oral surgeons have the same opinion on the role of the third molar in causing anterior crowding. The majority of both groups of clinicians do not consider their preventive extraction useful in order to prevent anterior crowding.

## Background

The relation between third molars and dental crowding has not yet been clarified in the literature. Clinicians have always been divided between supporters and opponents of anterior dental crowding produced by the force generated by the third molar eruption. For the same reason, the surgical prophylactic approach for the third molar has always been seen as the cure by the former and a ‘placebo’ by the latter.

Many articles are available about this topic in the literature. Bergstrom [1] in 1961 was one of the first authors to analyze the influence of the third molar in the developing dental arch and to say that there was a relationship between the teeth and the incisor change. Vego [2] 1 year later concluded that the eruption of lower third molars could exert a force on the neighboring teeth. More recently, Lindqvist [3] maintained that the eruption would create a pressure toward the anterior teeth.

On the contrary, Broadbent [4] was one of the first authors to support the opposite theory whereby the presence of third molars had no influence on the teeth. Many other authors reported no correlation between third molars and anterior crowding. Sidlauskas [5] and Richardson [6] did not consider the force exerted by the wisdom tooth capable of causing crowding. Southard [7] analyzed the eruption process and concluded that there is no force generated by that, and even if it existed, it would be insufficient to significantly affect anterior crowding. Karasawa [8] concluded that the presence of wisdom tooth had no influence on anterior teeth. Mettes et al. [9] in a systematic review showed that there was no sufficient evidence to support the prophylactic extraction theory. Bishara [10] from his systematic review concluded that the influence of the third molars on the alignment of the anterior dentition may be controversial, but there is no evidence to incriminate these teeth as being the only or even the most important etiologic factor in the post-treatment changes in incisor alignment. Marielle Blake et al. [11] from their review concluded that ‘if third molars were a contributing factor in the development of late lower incisor crowding, their role is likely to be one of minor importance’.

A randomized controlled trial was conducted by Harradine et al. [12] on 77 patients. They evaluated Little's index of irregularity, intercanine width, and arch length in patients after completion of orthodontic treatment randomly submitted to third molar extraction. The difference in crowding between the group with extracted third molar and the group with retained third molar was not clinically significant, and therefore, the removal of third molars to reduce or prevent late incisor crowding could not be justified.

Even if the recent literature available on this topic denies a correlation between third molar eruption and anterior incisor crowding [13,14], Lindauer et al. [15] in a survey between US practitioners identified significant differences in the mindset of oral surgeons and orthodontists. According to Lindauer, surgeons were still significantly more likely than orthodontists to believe that erupting third molars produce an anterior component of force and cause crowding of the anterior dentition, and were therefore more likely to recommend prophylactic removal of third molars to prevent crowding.

The aim of this work is to compare the current opinion of orthodontists and oral surgeons among the Italian practitioners.

## Methods

A six-question questionnaire was created using Google Chrome and was sent to some members of the Italian Society of Orthodontics (SIDO) and the Italian Society of Oral Surgery (SICOI).

The questions were:

1. Which category do you belong to? (orthodontist - oral surgeon).
2. How old are you? (<45 years / >45 years).
3. Do you think that the eruption of upper third molar is able to create anterior dental crowding? (yes/no) (always, often, sometimes, rarely).
4. Do you think that the eruption of lower third molar is able to create anterior dental crowding? (yes/no) (always, often, sometimes, rarely).
5. Do you consider the prophylactic extraction of the upper third molar useful to prevent anterior dental crowding? (yes/no) (always, often, sometimes, rarely).
6. Do you consider the prophylactic extraction of the lower third molar useful to prevent anterior dental crowding? (yes/no) (always, often, sometimes, rarely).

Institutional approval by both societies was granted. One hundred ninety-three (80 SICOI, 113 SIDO) members from either society from every region of Italy, participating in a national meeting held in Rome in 2013, were asked to answer to the online questionnaire. Information on the research objectives and how to complete the survey was given.

Respondents were informed, in the first part of the questionnaire, that by answering the survey, they consented to the use of that data.

Members had to indicate, choosing from a pop-up menu, their opinion on the role played by the third molar eruption in incisor crowding both in the lower and upper maxilla. They also had to report their clinical viewpoint on the effectiveness of third molar extraction in order to prevent dental crowding.

The survey was completely anonymous, and the researchers were not aware who sent or did not send the answers. Data were collected in an Excel document by MG and analyzed by a statistician (MM). Pearson's chi-square test was used to determine differences in responses between orthodontists and oral surgeons and to analyze whether there was a relationship between answers and practitioner's age.

## Results

A total of 166 members of both societies completed the online research survey. The overall response rate (RR) was 86%; 69 (41.6%) were oral surgeons (86.25% RR) and 97 (58.4%) were orthodontists (85.64% RR). Among them, 60.6% aged more than 45 years while 39.4% were younger.

No statistically significant differences were found in any of the questions submitted to the two groups.

The 83.8% of the surgeons and the 82.5% of the orthodontists consider the force generated by the upper third molar eruption not able to cause dental crowding. Lower percentages are reported in the mandible arch by the surgeons (63.8%) and orthodontists (52.6%). No statistically significant differences between surgeons and orthodontists were found regarding this question (Table 1).

**Table 1:** ‘**Do you think that the eruption of upper/lower third molar is able to create anterior dental crowding?’**

	**Orthodontists**	**Oral surgeons**
Maxilla, *P* = 0.82		
Yes	17 (17.5%)	11 (16.2%)
No	80 (82.5%)	57 (83.8%)
Mandible, *P* = 0.15		
Yes	47 (47.4%)	25 (36.2%)
No	50 (52.6%)	44 (63.8%)

Similar percentages were reported about the role of the third molar extraction to prevent dental crowding: 84.1% of the surgeons and 89.7% of the orthodontists do not consider the upper third molar extraction useful, while 63.2% of the surgeon and the 58.8% of the orthodontists do not consider the lower third molar extraction useful (Table 2)*.*

**Table 2:** **‘Do you consider the prophylactic extraction of the upper/lower third molar useful to prevent anterior dental crowding?’**

	**Orthodontists**	**Oral surgeons**
Maxilla, *P* = 0.28		
Yes	10 (10.3%)	11 (15.9%)
No	87 (89.7%)	58 (84.1%)
Mandible, *P* = 0.56		
Yes	40 (41.2%)	26 (36.8%)
No	57 (58.8%)	43 (63.2%)

In dealing with the upper arch in both groups, almost all the clinicians agree in avoiding third molar extraction; only 10.3% of orthodontists and 15.9% of oral surgeons consider this practice useful to prevent upper incisor crowding. Similar percentages are reported on the relationship between eruption and crowding: 16.2% of the surgeons think that in the maxilla there is a relationship, but 6 (50%) of them answered ‘sometimes’, 2 (16.7%) ‘often’, 4 (33.3%) ‘rarely’, and nobody answered ‘always.’ The percentage between orthodontists was similar: 17.5% maintain that in the maxilla, the force due to third molar eruption is able to create anterior crowding, but 6 (31.6%) of them answered ‘sometimes’, 2 (10.5%) ‘often’, 11 (57.9%) ‘rarely’, and again nobody said ‘always’.

The results show that the majority of practitioners irrespective of their specialization think that in the upper arch, the force is not capable of causing dental crowding.

On the contrary, the results related to the mandible arch show a higher percentage of surgeons (36.2%) and orthodontists (47.4%) thinking that the produced force is able to generate crowding; 60% of surgeons answered ‘sometimes,’ 20% ‘often’, 16% ‘rarely’, and 4% ‘always’, while among orthodontists, 66% answered ‘sometimes’, 19.1% ‘often’, 10.6% ‘rarely’, and 4.3% ‘always’. These results show a different mindset about the role played by the lower third molar; practitioners are approximately divided in two equal groups between supporters and opponents of the ‘lower third molar crowding theory’ always irrespective of their specializations even if results are even more striking for orthodontists.

Answers were also analyzed according to clinicians' age. The results are shown in Tables 3 and 4. A total of 100 practitioners (60.6%) were older than 45 years old, while 65 were younger than 45 years old.

**Table 3:** ‘**Do you think that the eruption of upper/lower third molar is able to create anterior dental crowding?’**

	**Orthodontists**		**Oral surgeons**	
	**<45 years**	**>45 years**	**<45 years**	**>45 years**
Maxilla				
Yes	6 (16.2%)	11 (18.6%)	2 (7.1%)	9 (22.5%)
No	31 (87.8%)	48 (81.4%)	26 (92.9%)	31 (77.5%)
Mandible				
Yes	17 (45.9%)	17 (41.5%)	8 (28.6%)	17 (41.5%)
No	20 (54.1%)	24 (58.5%)	20 (71.4%)	24 (58.5%)

Answers according to age groups, *P* > 0.05.

**Table 4:** ‘**Do you consider the prophylactic extraction of the upper/lower third molar useful to prevent anterior dental crowding?’**

	**Orthodontists**		**Oral surgeons**	
	**<45 years**	**>45 years**	**<45 years**	**>45 years**
Maxilla				
Yes	4 (10.8%)	6 (10.1%)	2 (7.1%)	9 (22%)
No	33 (89.2%)	53 (89.9%)	26 (92.9%)	32 (78%)
Mandible				
Yes	15 (40.5%)	25 (42.4%)	7 (25.9%)	18 (43.9%)
No	22 (59.5%)	34 (57.6%)	20 (74.1%)	23 (56.1%)

Answers according to age groups, *P* > 0.05.

Forty-one (59.4%) surgeons were older than 45 years old and 28 (40.6%) were younger than 45 years old, while 59 (61.5%) orthodontists were older than 45 years old and 37 (38.1%) younger than 45 years old. There were no statistically significant differences between groups (*P* > 0.005) even if a slight difference was observed between the two age categories especially among oral surgeons.

## Discussion

Even if the recent literature had clarified the marginal role of third molar eruption in the genesis of anterior crowding, this topic continues to be controversial among clinicians.

Orthodontists are generally considered more conservative and more used to retain healthy wisdom teeth and not considering them a cause of incisor crowding; oral surgeons, on the other hand, usually have a more interventionist approach leading to the extraction of all the four wisdom teeth even if asymptomatic.

The survey did not show any statistically significant differences in the answers between the two groups (surgeons vs. orthodontists), but it pointed that a considerable part of Italian practitioners still think that crowding is linked to the third molar eruption. Even if the majority of Italian orthodontists do not believe in this relationship and responded ‘no’, there is still a great percentage of ‘yes’ especially dealing with the mandible (47.4%).

This is in accordance with the study conducted by Lindauer et al. [15] on US practitioners using a similar questionnaire. In fact, they showed that more than half of the US orthodontists and oral surgeons consider the force generated by the lower third molar eruption capable of generating anterior crowding.

Lindauer et al., on the contrary, founded a statistically significant difference between clinicians; oral surgeons were more likely than orthodontists to retain the third molar which is capable of causing incisor crowding. Curiously, the opinion of Italian and US practitioners, especially between oral surgeons, is almost the opposite, with the Italians being more likely not to support the theory of crowding. For example, 78.2% of the US surgeons vs. 36.2% of the Italian consider the mandibular third molar capable of causing incisor crowding.

Similar results were reported by Tüfekçi et al. [16] in a study analyzing opinions of Swedish and US orthodontists. Also in this study, more than half of Swedish orthodontists (65%) consider the lower third molar capable of causing incisor crowding.

The second set of questions was about the extraction of healthy third molar teeth as prophylactic treatment to prevent anterior crowding. It is important to underline that neither the National Institute of Clinical Excellence (NICE) in 2000 [17] nor the Scottish Intercollegiate Guidelines Network (SIGN) in 1999 reviewed in 2005 [18] considered potential tertiary crowding as a reason to justify the prophylactic extraction of third molars. They concluded that given the costs and risks associated with third molar extractions, there was no valid evidence to support the prophylactic removal of pathology-free (asymptomatic) third molars [19]. A recent review on asymptomatic third molars concluded that it could be more logical to just monitor these teeth over time rather than extract them [20].

In this study, the majority of orthodontists and surgeons believe that prophylactic extraction is not useful both in the upper and in the lower arch to prevent incisor crowding: similar percentages are reported for the maxilla (89.7% orthodontists, 81.4% surgeons) and for the mandible (58.8% orthodontists, 63.2% surgeons).

The surprising element of this research is the position of oral surgeons, who have always been considered less conservative than orthodontists; in Italy, they currently agree in not suggesting the prophylactic extraction. Lindauer et al., on the contrary, found that the majority of orthodontists believe that the extraction is ‘never’ useful neither in the upper nor in the lower arch, while surgeons are more likely to suggest the extraction of the teeth.

Another variable investigated was the ages of the practitioners included in the survey. The decision to investigate a possible relationship between age and answers is based on the theory that older practitioners are more likely than the younger ones to think that the third molar can cause crowding. However, the results show no significant differences between the groups. Both agree to think that the forces expressed by the teeth are unable to create incisor movement, and according to such results, they do not recommend prophylactic extraction.

It is interesting to see that the opinions of the younger orthodontists do not differ from those of the older orthodontists; on the contrary, there is a difference between younger and older surgeons. Although not statistically significantly different, the younger are more likely to not suggest third molar extraction both in the upper (92.9%) and lower arch (74.1%) in contrast with the older (78% and 56.1%, respectively).

## Conclusions

The influence of third molars on incisor crowding, denied by the recent literature, remains controversial between clinicians. Considering the limits of the present survey, in particular the small size of the groups, no statistically significant differences were observed between Italian oral surgeons and orthodontists. The majority of Italian orthodontists and oral surgeons consider the upper third molar not able to cause dental crowding. On the other hand, contrasting percentages are reported for the lower third molar; especially, orthodontists are divided between supporters and opponents of the theory of the lower third molar as a cause of crowding. Both groups do not recommend the upper third molar extraction to prevent anterior crowding, but are more likely to suggest lower third molar extraction. Finally, no statistically significant differences were reported in relation to practitioners' age.

## References

[1] Bergstrom K, Jensen R. **Responsibility of the third molar for secondary crowding.** *Dent Abstr.* 1961; **6:**544.

[2] Vego L (1962). A longitudinal study of mandibular arch perimeter. Angle Orthod.

[3] Lindqvist B, Thilander B (1982). Extraction of third molars in cases of anticipated crowding in the lower jaw. Am J Orthod.

[4] Broadbent BH (1941). Ontogenic development of occlusion. Angle Orthod.

[5] Sidlauskas A, Trakiniene G (2006). Effect of the lower third molars on the lower dental arch crowding. Stomatologija.

[6] Richardson ME (1982). Late lower arch crowding in relation to primary crowding. Angle Orthod.

[7] Southard TE, Weeda LW (1991). Mesial force from unerupted third molars. Am J Orthod.

[8] Karasawa LH (2013). Cross-sectional study of correlation between mandibular incisor crowding and third molars in young Brazilians. Med Oral Patol Oral Cir Bucal.

[9] Mettes TD, Ghaeminia H, Nienhuijs ME, Perry J, van der Sanden WJ, Plasschaert A: **Surgical removal versus retention for the management of asymptomatic impacted wisdom teeth.** *Cochrane Database Syst Rev*. 2012; (Issue 6):CD003879. 10.1002/14651858.CD003879.pub3 22696337

[10] Bishara SE (1999). Third molars: a dilemma! Or is it?. Am J Orthod Dentofacial Orthop.

[11] Blake M, Bibby K (1998). Retention and stability: a review of the literature. Am J Orthod Dentofacial Orthop.

[12] Harradine NW, Pearson MH, Toth B (1998). The effect of extraction of third molars on late lower incisor crowding: a randomized controlled trial. Br J Orthod.

[13] Zachrisson BU (2005). Mandibular third molars and late lower arch crowding–the evidence base. World J Orthod.

[14] Hasegawa Y, Terada K, Kageyama I, Tsuchimochi T, Ishikawa F, Nakahara S (2013). Influence of third molar space on angulation and dental arch crowding. Odontology.

[15] Lindauer SJ, Laskin DM, Tüfekçi E, Taylor RS, Cushing BJ, Best AM (2007). Orthodontists' and surgeons' opinions on the role of third molars as a cause of dental crowding. Am J Orthod Dentofacial Orthop.

[16] Tüfekçi E, Svensk D, Kallunki J, Huggare J, Lindauer SJ, Laskin DM (2009). Opinions of American and Swedish orthodontists about the role of erupting third molars as a cause of dental crowding. Angle Orthod.

[17] NICE (2000). Guidance on the Extraction of Wisdom Teeth.

[18] SIGN (1999). Management of Unerupted and Impacted Third Molar Teeth.

[19] Kandasamy S, Rinchuse DJ, Rinchuse DJ (2009). The wisdom behind third molar extractions. Aust Dent J.

[20] Mettes TG, Nienhuijs ME, van der Sanden WJ, Verdonschot EH, Plasschaert AJ: **Interventions for treating asymptomatic impacted wisdom teeth in adolescents and adults.** *Cochrane Database Syst Rev*. 2005; (Issue 2):CD003879. 10.1002/14651858.CD003879.pub2 15846686

